# Sub-Inhibitory Doses of Individual Constituents of Essential Oils Can Select for *Staphylococcus aureus* Resistant Mutants

**DOI:** 10.3390/molecules24010170

**Published:** 2019-01-04

**Authors:** Daniel Berdejo, Beatriz Chueca, Elisa Pagán, Adriana Renzoni, William L. Kelley, Rafael Pagán, Diego Garcia-Gonzalo

**Affiliations:** 1 Tecnología de los Alimentos, Instituto Agroalimentario de Aragón-IA2 (CITA-Universidad de Zaragoza), Miguel Servet 177, 50013 Zaragoza, Spain; berdejo@unizar.es (D.B.); bchuecaomella@gmail.com (B.C.); epagan@unizar.es (E.P.); pagan@unizar.es (R.P.); 2 Service of Infectious Diseases, Department of Medical Specialties, University Hospital of Geneva, 1205 Geneva, Switzerland; Adriana.Renzoni@unige.ch; 3 Department of Microbiology and Molecular Medicine, University Hospital and Medical School of Geneva, 1205 Geneva, Switzerland; william.kelley@unige.ch

**Keywords:** whole genome sequencing, genotypic resistance, carvacrol, citral, (+)-limonene oxide

## Abstract

Increased bacterial resistance to food preservation technologies represents a risk for food safety and shelf-life. The use of natural antimicrobials, such as essential oils (EOs) and their individual constituents (ICs), has been proposed to avoid the generation of antimicrobial resistance. However, prolonged application of ICs might conceivably lead to the emergence of resistant strains. Hence, this study was aimed toward applying sub-inhibitory doses of the ICs carvacrol, citral, and (+)-limonene oxide to *Staphylococcus aureus* USA300, in order to evaluate the emergence of resistant strains and to identify the genetic modifications responsible for their increased resistance. Three stable-resistant strains, CAR (from cultures with carvacrol), CIT (from cultures with citral), and OXLIM (from cultures with (+)-limonene oxide) were isolated, showing an increased resistance against the ICs and a higher tolerance to lethal treatments by ICs or heat. Whole-genome sequencing revealed in CAR a large deletion in a region that contained genes encoding transcriptional regulators and metabolic enzymes. CIT showed a single missense mutation in *aroC* (N187K), which encodes for chorismate synthase; and in OXLIM a missense mutation was detected in *rpoB* (A862V), which encodes for RNA polymerase subunit beta. This study provides a first detailed insight into the mechanisms of action and *S. aureus* resistance arising from exposure to carvacrol, citral, and (+)-limonene oxide.

## 1. Introduction

Emergence of stable resistant strains during food processing can compromise food safety and shelf-life. Development of stable bacterial resistance is based on genetic modifications caused by mutations (i.e., genotypic resistance) [1] that produces variations in cellular responses to stress and damage [2]. Mutated microorganisms with an elevated resistance and/or tolerance might survive food preservation treatments that were previously considered sufficient as a control measure for spoiling. Thus, in recent years many efforts have been carried out in the search for new food preservatives capable of avoiding the generation of antimicrobial resistance.

Many authors have proposed the use of natural compounds, such as essential oils (EOs) and their individual constituents (ICs) as food preservatives. Carvacrol, citral, and (+)-limonene oxide are monoterpenes generally recognized as safe (GRAS), whose antimicrobial properties have been demonstrated [3] and their mechanisms of microbial inactivation, mainly targeting cell envelopes, have been extensively studied [4,5,6]. However, the complete picture of the effects of these compounds over the bacterial cells is not fully understood.

Although it has been generally accepted that EOs and ICs do not have mutagenic properties [7,8], hyper-resistant strains of *Escherichia coli* have been isolated after bacterial exposure to carvacrol, citral, and (+)-limonene oxide during growth [9]. Rather than increasing the mutation rate, the presence of these compounds would create a selective pressure which leads to the emergence of mutant strains displaying resistance [9]. Due to the relevance of those results, it is convenient to determine the effect of exposure to sub-inhibitory concentrations of these natural compounds in the isolation of resistant mutants in pathogenic bacteria, such as the Gram-positive *Staphylococcus aureus* or *Listeria monocytogenes*. In addition, whole genome sequencing (WGS) of mutant strains would allow the identification of the precise genetic modifications in comparison to the wild type (WT) strain. This information might assist in the description of the mechanism of cell response to these antimicrobial compounds, providing valuable information for the design of more suitable food preservation methods. Accordingly, WGS of a resistant *E. coli* strain isolated from an evolution experiment in the presence of carvacrol revealed a relevant role of *soxR* in bacterial tolerance against the same IC used for selection, i.e., carvacrol (direct-tolerance), and other ICs, such as citral and (+)-limonene oxide (cross-tolerance) [10].

Thus, the aims of this study were (a) to isolate stable mutant resistant strains of *Staphylococcus aureus* USA300 by applying sub-inhibitory doses of carvacrol, citral, and (+)-limonene oxide during bacterial growth; (b) to characterize their resistance and survival against different food preservation technologies; and (c) to identify the genetic modification(s) associated with their increase of resistance and tolerance.

## 2. Results

### 2.1. Isolation of S. aureus USA300 Derivative Strains with Increased Resistance to ICs

In order to carry out the selection of stable-resistant strains by exposing *S. aureus* USA 300 to sub-inhibitory concentrations of ICs during bacterial growth, the minimum inhibitory concentration (MIC) of WT strain against carvacrol, citral, and (+)-limonene oxide was first determined (Table 1). Thus, 50 µL/L of carvacrol, 75 µL/L of citral, or 375 µL/L of (+)-limonene oxide (½ × MIC) were added to growth media for strain isolation. After a 10-day evolution experiment, six colonies from each culture were randomly selected, isolated, and re-cultured without any ICs. Hereinafter, the strains isolated after carvacrol exposure were referred to as CAR, those isolated after citral exposure as CIT, and after (+)-limonene oxide as OXLIM. The *S. aureus* USA300 WT strain was used as a reference to evaluate variations on the resistance of derivative strains by the disk diffusion assay and the MIC determination. The six colonies selected from each culture showed similar levels of resistance against the three ICs (*p >* 0.05; data not shown). Therefore, one colony from each culture was selected for further analyses. As shown in Table 1, derivative strains showed an increased resistance (*p* ≤ 0.05) against the IC used in the evolution experiment (i.e., direct-resistance). The CAR strain showed an MIC against carvacrol of 150 µL/L, which represents an increase of 50% compared to WT. Similarly, CIT and OXLIM strains showed 65 and 100% increases in MIC against citral and (+)-limonene oxide, respectively. These increases in resistance were also observed using a disk diffusion assay (Table S1).

Additional analysis revealed that the CAR strain showed similar MIC levels against citral and (+)-limonene oxide compared with WT, whereas CIT and OXLIM showed cross-resistance against the other ICs tested (Table 1).

### 2.2. Effect of ICs on Mutation Frequency during Bacterial Growth 

**Figure 1:**
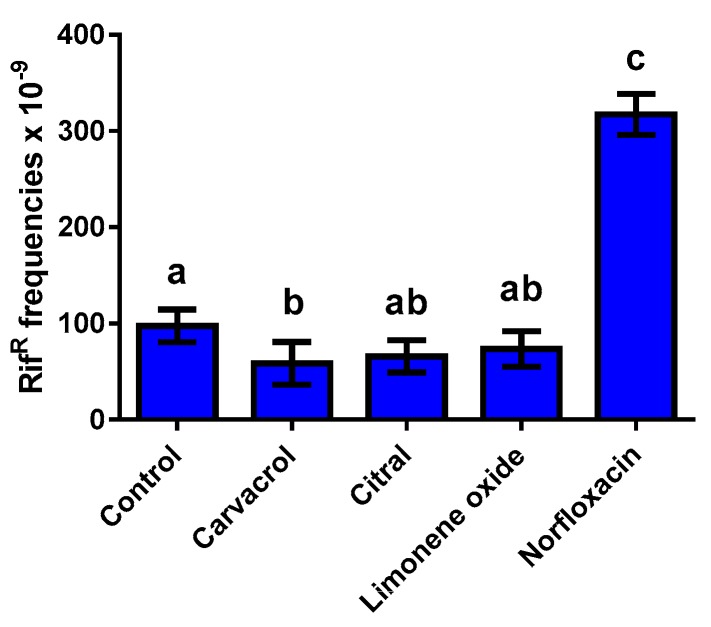
Mutagenesis frequency in *S. aureus* USA300 grown in broth without (control) and with carvacrol (50 μL/L), citral (75 μL/L), (+)-limonene oxide (375 μL/L), and norfloxacin (2000 mg/L). Mutagenesis frequency was expressed as rifampicin-resistant cells in the total microbial population. Data are means ± standard deviations (error bars) obtained from five independent experiments. Letters over the bars represent statistically significant differences; different lower-case letters above the bars represent statistically different values (*p* ≤ 0.05), while results with the same letter show no significant difference (*p* > 0.05).

The role of ICs in the emergence of resistance was evaluated by the determination of mutation rates, specifically mutations leading to resistance against rifampicin [11]. We evaluated the mutation frequency during bacterial growth without (control) and with ½ × MIC of each IC or with norfloxacin at 2000 mg/L. As shown in Figure 1, the WT strain showed a spontaneous frequency of rifampicin-resistant mutants over 100 × 10^–9^ during bacterial growth in absence of ICs. The incubation of WT strain in the presence of norfloxacin increased the emergence of rifampicin-resistant mutants 3-fold. However, no significant differences (*p >* 0.05) were observed in the mutation rate in the presence of citral or (+)-limonene oxide. Moreover, we observed that the mutation frequency was reduced in presence of carvacrol, with a mutation rate of approx. 60 × 10^–9^ (*p* < 0.05). Collectively, we conclude that the exposure of USA300 to ICs did not alter the mutation rate under our experimental conditions.

### 2.3. Evaluation of Derivative Strains against Lethal Treatments 

**Figure 2:**
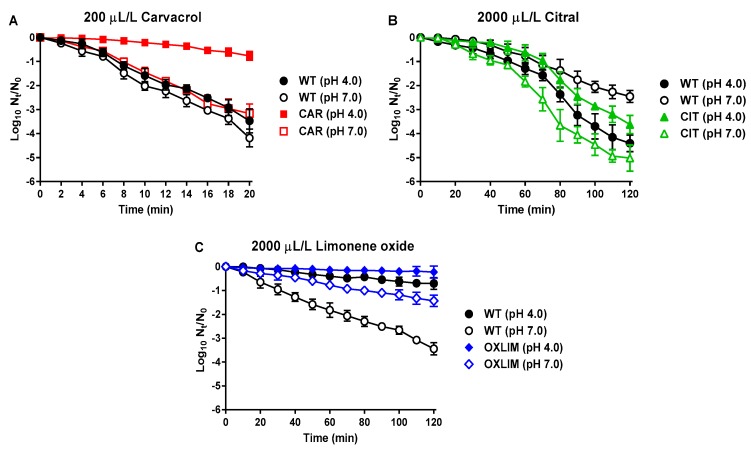
Inactivation of *Staphylococcus aureus* USA300 wild type (WT, 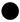,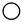) and its derivative strains; CAR ( 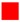,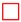) using 200 μL/L carvacrol (**A**), CIT (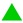,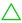) using 2000 μL/L citral (**B**), and OXLIM (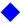,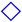) using 2000 μL/L (+)-limonene oxide (**C**) treatment, at pH 4.0 (black symbols) and pH 7.0 (white symbols). All treatments were performed at room temperature except (+)-limonene oxide, which were carried out at 37 °C. Data are means ± standard deviations (error bars) obtained from at least three independents experiments.

After determination of the increased resistance of derivative strains, their tolerance against lethal treatments with ICs, heat, and pulsed electric fields (PEF) was determined. First, we evaluated the death of WT and derivative strains caused by the same IC used in their selection protocol (i.e., direct-tolerance, Figure 2). Survival at neutral and acid pH was evaluated. As shown in Figure 2A, CAR was more tolerant against a carvacrol lethal treatment than WT strain at pH 4.0: after 20 min, inactivation of WT cells was 3 log_10_ cycles higher than that of CAR cells. Differences in inactivation by carvacrol at pH 7.0 between WT and CAR were less relevant, showing a difference of 1 log_10_ cycles of inactivation only after 20 min of treatment.

Although a slight increase in survival of CIT cells was detected after citral treatments at pH 4.0, a higher inactivation of CIT cells was determined at pH 7.0 in comparison to WT cells (Figure 2B): after 120 min with citral at pH 7.0, a microbial reduction of 2.5 log_10_ cycles of initial population of WT was achieved in contrast to 5 log_10_ cycles of CIT cells. While survival to carvacrol and citral treatments was evaluated at room temperature, higher temperature was needed to cause bacterial inactivation with (+)-limonene oxide. Therefore, the temperature was raised to 37 °C to carry out the lethal treatments of (+)-limonene oxide. A higher survival of OXLIM was demonstrated after (+)-limonene oxide treatment at pH 7.0, but not at pH 4.0 (Figure 2C), as compared to WT survival under the same conditions. An increase in tolerance with regard to WT against the IC used for selection was higher for CAR at pH 4.0 than for CIT at pH 7.0.

**Figure 3:**
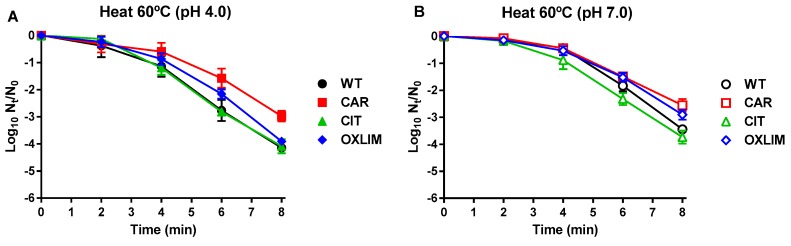
Inactivation of *Staphylococcus aureus* USA300 wild type (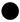,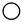) and its derivative strains: CAR (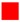,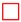), CIT (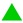,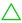), and OXLIM (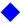,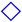) using heat treatments (60 °C) at pH 4.0 (A, black symbols) and pH 7.0 (B, white symbols). Data are means ± standard deviations (error bars) obtained from at least three independents experiments.

Cross-tolerance of derivative strains against physical food preservation technologies, such as heat and PEF treatments, was also evaluated. Figure 3 shows inactivation of WT and the derivative strains after a heat treatment at 60 °C in a buffer of pH 4.0 (Figure 3A) or 7.0 (Figure 3B). No significant differences were detected among the survival of WT, CIT, and OXLIM after heat treatments at pH 4.0 (Figure 3A). Only CAR showed a significantly higher survival than WT (*p* ≤ 0.05) under these conditions: while inactivation of CAR after 8 min of heat treatment was approx. 3 log_10_ units, initial population of WT, CIT, and OXLIM was reduced in approx. 4 log_10_ units. With regard to heat treatments at pH 7.0, no differences were found between WT and CIT (*p* > 0.05), with a population reduction of 3.6 log_10_ units after 8 min (Figure 3B). However, in comparison to WT, an increased survival of CAR and OXLIM after the same treatment was detected (*p* ≤ 0.05), with 2.5 and 2.9 log_10_ units of inactivation, respectively.

**Figure 4:**
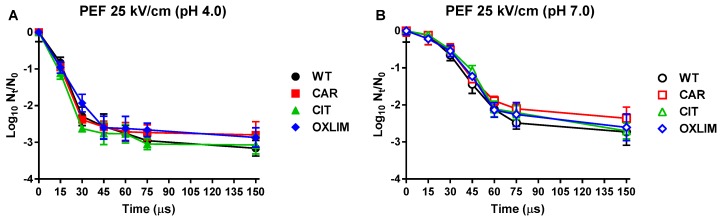
Inactivation of *Staphylococcus aureus* USA300 wild type (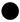,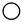) and its derivative strains: CAR (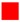,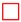), CIT (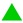,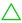), and OXLIM (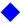,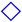) using pulsed electric fields treatments (PEF: 25 kV/cm, 1 Hz, 3 µs/pulse) at pH 4.0 (**A**, black symbols) and pH 7.0 (**B**, white symbols). Data are means ± standard deviations (error bars) obtained from at least three independents experiments.

Figure 4 represents inactivation of WT and derivative strains caused by PEF treatments of 25 kV/cm, at pH 4.0 (Figure 4A) and 7.0 (Figure 4B). PEF treatments at pH 4.0 for 60 μs or longer caused approx. 3 log_10_ cycles of inactivation of WT, being more effective than at pH 7.0. Derivative strains showed a similar survival to PEF treatments (*p* > 0.05) in comparison to WT at both pH, indicating the absence cross-tolerance to PEF of the derivative strains. 

**Figure 5:**
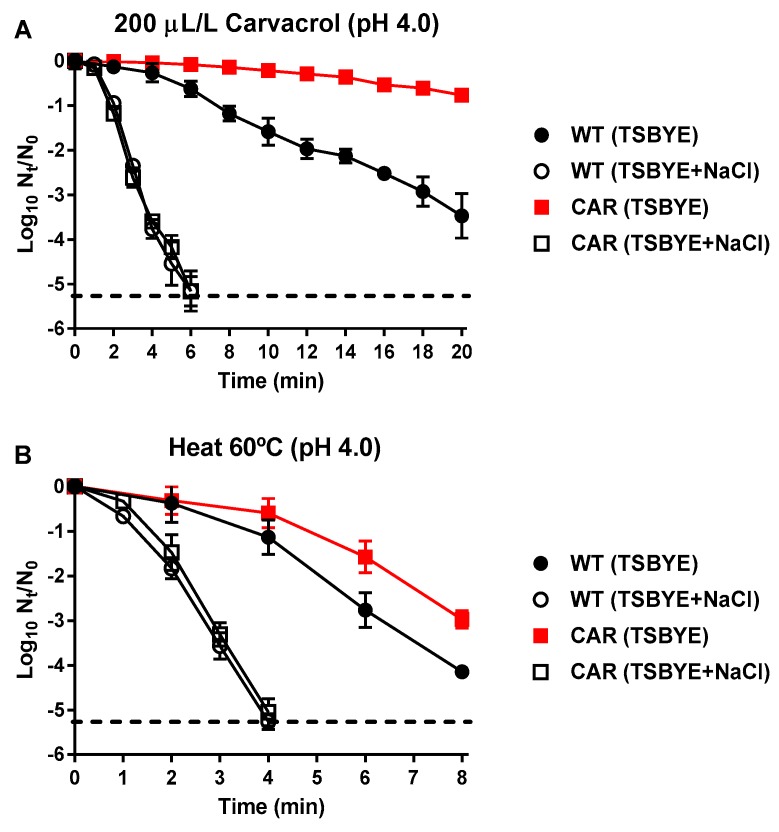
Inactivation of *Staphylococcus aureus* USA300 wild type (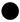,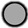) and CAR derivative strain (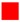,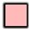) using carvacrol (200 μL/L; **A**) and heat treatments (60 °C; **B**) at pH 4.0. The treated samples were recovered in TSBYE without (black symbols) and with 14% of NaCl (semi-transparent symbols), used as selective agent to detect sublethal damage. Data are means ± standard deviations (error bars) obtained from at least three independents experiments. Discontinuous line shows the detection limit.

Considering these results on survival to lethal treatments, CAR was shown to be the most tolerant strain. Therefore, this strain was selected to determine the occurrence of sublethal injuries in the cytoplasmic membrane after these treatments, in order to determine whether the increased survival was related to an increased intrinsic cell tolerance or an improved damage-repair system. CAR and WT were treated with carvacrol or heat at pH 4.0 (Figure 5), the pH at which the greater differences in tolerance between both strains were observed. Treated cells were plated in non-selective and selective media with NaCl added. The latter medium would avoid the growth of damaged cells in the cytoplasmic membrane and only permit the growth of intact cells [12]. Results from carvacrol (Figure 5A) and heat (Figure 5B) treatments revealed that although microbial counts were higher for CAR than for WT in the non-selective recovery medium, no significant differences (*p >* 0.05) were observed when samples were recovered in the selective medium.

### 2.4. Genomic Sequencing of WT and Derivative Strains

In order to identify metabolic pathways and/or key structures involved in the increased resistance and survival of the derivative strains, WGS of these strains was conducted. Since WT, CAR, CIT, and OXLIM are derivatives of *S. aureus* USA300 (NCBI accession: NC_007793.1), this strain was used as a reference to evaluate genome coverage and facilitate contig assembly. The number of reads in whole-genomic sequencing were 17,311,886, 17,263,646, 13,788,036, and 16,852,412-bp paired-end reads for WT, CAR, CIT, and OXLIM, respectively, mapping the reference genome. Mapping covered 15,853,227, 16,399,422, 13,081,387, and 16,147,625 bases in WT, CAR, CIT, and OXLIM, resulting in 91.6%, 95.0%, 94.9%, and 95.8% of *S. aureus* USA300 genome coverage (2,872,769 bases). Since we sequenced the *S. aureus* USA300 strain that was the isogenic precursor of CAR, CIT, and OXLIM strains, differences were only extracted between our laboratory strain (WT) and ICs derivatives.

**Figure 6:**
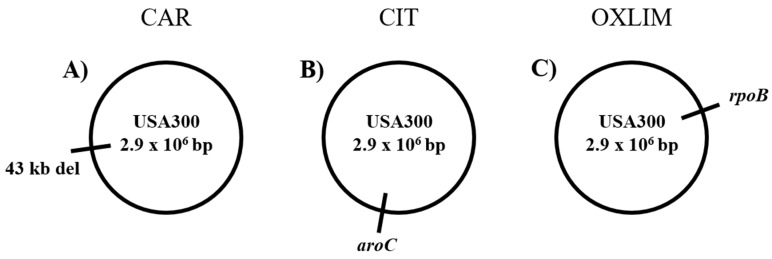
Genomic maps of the *Staphylococcus aureus* USA300 derivative strains from carvacrol (CAR; **A**), citral (CIT; **B**), and (+)-limonene oxide (OXLIM; **C**) chromosomes.

After assembling the genomes of the four strains, computer analysis revealed six mutations in CAR (3 single nucleotide polymorphisms (SNPs), 2 insertions or deletions (InDels), and 1 large deletion), 4 in CIT (3 SNPs and 1 InDels), and 7 in OXLIM (5 SNPs and 2 InDels) compared to WT strain. In order to confirm these genetic differences, specific primers (Table S2) were designed to examine the region of each mutation using PCR amplification using gDNA and Sanger sequencing of the amplified fragments. The confirmed mutations are depicted on circular schematic maps (Figure 6).

First, only the large deletion in CAR was confirmed (Table 2), while the other mutations were rejected after PCR confirmation. This large deletion was located from 2,084,675 to 2,127,700 bp (genome position) with a size of 43,025 bp. Consequently, 65 genes were deleted in CAR (Table S3). 

With regard to CIT and OXLIM, only one SNP in each strain was verified (Table 2), and the remaining genetic differences detected using computer analysis of WGS were rejected. SNP verified in CIT was located in genome position 1,524,394 bp, changing adenine to thymine (transversion) in the *aroC* gene, resulting in a missense mutation AroC N187K. In OXLIM a SNP was detected in *rpoB* (genome position 588,138 bp), in which cytosine was replaced by a thymine (transition). Conceptual translation predicted a missense mutation RpoB A862V.

## 3. Discussion

The use of EOs and their ICs as food preservatives has been proposed due to their antimicrobial properties [3,7]. In addition, recent studies have investigated the use of these compounds as therapeutics for infectious diseases to avoid the emergence of resistant bacteria against antibiotics [13,14]. Although several studies have discarded the induction of stable bacterial resistance by the application of EOs and ICs [8,15,16,17], a recent study has demonstrated the emergence of hyper-resistant strains after exposure to carvacrol, citral, and (+)-limonene oxide in a Gram-negative bacteria, *E. coli* MG1655 [9]. Following the same protocol, in this study we have isolated mutant strains with increased resistance and tolerance of a Gram-positive bacteria, *S. aureus* USA300, after the exposure during bacterial growth in presence of ICs at sub-inhibitory concentrations (Table 1).

It is generally acknowledged that bacterial exposure to sub-lethal levels of antibiotics leads to emergence of antibiotic resistant strains; for example, fluoroquinolones and β-lactams increase the mutagenesis rate, thereby accelerating the generation of resistant strains [18]. In agreement with previous studies on ICs and EOs in *E. coli* MG1655 [9] and *S. aureus* ATCC 25923 [19], carvacrol, citral, and (+)-limonene oxide did not increase mutation frequency in *S. aureus* USA300 (Figure 1). As expected, nor increased the mutation frequency under the same growth conditions (Figure 1). Sublethal antibiotic treatments increase intracellular reactive oxygen species (ROS) production that damages DNA and activates SOS response and RecA activity [1,20]. This mechanism leads to bacterial multidrug resistance via induced mutagenesis [18]. Although ROS are also involved in the mechanism of bacterial death by carvacrol, citral, and (+)-limonene oxide [4,5], a lower mutagenesis was induced by these antimicrobial compounds. Since these natural compounds also display strong antioxidant properties [21], they might alleviate oxidative stress, thereby protecting cells from DNA damage, which would decrease the mutagenesis rate. 

After selecting one mutant strain from each evolution experiment (one with each IC), further sensitivity experiments against food preservation treatments were carried out. There are two bacterial strategies to survive under the presence of antimicrobials compounds as a function on the compound concentration and treatment duration: resistance and tolerance. While resistance is defined as the capability of bacteria to grow in the presence of an antimicrobial compound for a prolonged time, tolerance is a property of bacterial cells to survive to a high concentration of antimicrobial agent for a short time treatment [22]. Bacterial resistance is usually evaluated through bacteriostatic tests, such as MIC test, whereas tolerance determination requires an evaluation of survival against lethal treatments [23].

First, we demonstrated that the three strains isolated through the selective pressure of ICs had increased their MIC compared to WT. In addition, these strains did not only show a higher resistance against the ICs used in its selection, but also CIT and OXLIM displayed cross-resistance against the other ICs used in the selection protocol (Table 1). Next, we evaluated the survival of these resistant strains after lethal conditions (i.e., tolerance) with ICs and physical treatments (such as heat and PEF). As previously demonstrated in *S. aureus* [24,25], carvacrol and citral showed a strong bactericidal activity at room temperature against *S. aureus* USA300, causing a 4 log_10_ reduction of the initial population after 20 min at pH 4.0 with carvacrol and after 120 min at pH 7.0 with citral (Figure 2A,B). CAR and OXLIM showed an increased tolerance to lethal treatments of carvacrol and (+)-limonene oxide, respectively, compared to WT (Figure 2A,C). On the other hand, although CIT displayed an increased resistance in MIC, it was less tolerant than the WT strain to a lethal treatment using citral at pH 7 (Figure 2B). 

Regarding cross-tolerance, inactivation caused using heat and PEF treatments is shown in Figure 3; Figure 4 respectively. On the one hand, CAR showed an increase of heat tolerance compared to WT at both treatment pHs, whereas OXLIM only displayed a rise in tolerance against heat treatments at pH 7.0. No significant differences (*p* > 0.05) were observed in microbial counts after heat treatments between WT and CIT (Figure 3). Even though heat treatments lead to DNA and RNA damage, ribosome destabilization, and enzyme and other proteins denaturation, among others, it is acknowledged that the principal structure affected by heat treatments is the cell envelope [12,26,27]. Hence, it is expected that mutations of CAR and OXLIM are involved in tolerance of the cell membrane or its reparation system. On the other hand, the cytoplasmic membrane is the main target of PEF treatments, causing pores in its surface (electroporation), and consequently, causing bacterial inactivation [28,29,30]. Although ICs and PEF treatments mainly target the cell envelopes, derivative strains (CAR, CIT, and OXLIM) showed a tolerance against PEF treatment similar to WT (Figure 4). It is likely that ICs and PEF target different structures and/or metabolic pathways in the cell envelopes, as previously shown by the absence of synergistic lethal effects in combined processes with ICs and PEF [31,32].

Our results indicate that mechanisms of resistance against ICs are different from those involved tolerance to ICs. According to Brauner, Fridman, Gefen, and Balaban [22], the phenomena of tolerance and resistance are mechanistically distinct and assumed to be unrelated. Recent studies have reported that antibiotic tolerance precedes and facilitates the evolution of resistance because tolerance enhances the chances for resistance mutations to disseminate in the bacterial population [33,34]. However, the resistant strains obtained in this study (CAR, CIT, and OXLIM) were not always tolerant to ICs. Further studies are necessary to evaluate the appearance of resistance and tolerance to ICs.

With the aim of discerning whether the increased survival of derivative strains was related to an increased intrinsic tolerance of the cell or to a better recovery from damages caused by heat or ICs, the occurrence of sublethally injured cells in the cytoplasmic membrane was evaluated. Evaluation of sublethal damage was performed in the CAR strain after a carvacrol or a heat lethal treatment at pH 4.0, the conditions under the highest differences of tolerance were observed between WT and CAR. Similar survival curves were obtained after recovery of carvacrol- or heat-treated cells of CAR and WT in the selective media (Figure 5), indicating that the cytoplasmic membrane of both strains had the same intrinsic tolerance against heat and carvacrol [12]. Consequently, the higher microbial counts of treated CAR cells in a non-selective medium with regard to WT (Figure 5) would be due to an increased ability of CAR to repair the sublethal damages in its cytoplasmic membrane caused by heat or carvacrol. 

Thus, in order to identify the molecular changes involved in the higher tolerance/resistance of the derivative strains, whole genome sequencing of WT, CAR, CIT, and OXLIM was carried out. First, the only genetic modification in CAR (Table 2) was a large deletion with a size of 43,025 bp, where 65 genes were involved. Most of these genes are involved in phage cycles, DNA replication or code for transcriptional regulators and enzymes involved in energy production and conversion [35]. The genes within the deletion must be non-essential or compensated for by redundant functions provided by genes elsewhere in the genome. Therefore, one or more of these deleted genes were the cause of the increase of carvacrol-resistance and carvacrol- and heat-tolerance of CAR. According to Chueca et al. [36], 61 genes in *E. coli* were upregulated during a carvacrol treatment, coding genes for DNA-binding transcriptional regulator and genes related with phage shock response. In addition, transcription of DNA-binding protein (H-NS and the 50S ribosomal proteins L7/L12) was downregulated by thymol, an IC similar to carvacrol, leading to bacterial DNA stability and inhibition of transcription as a protective mechanism [37]. Thus, deletion of several genes involved in different metabolic processes in CAR, such as coding genes of DNA-binding protein, replication protein DnaD, or Xenobiotic Response Element family transcriptional regulator, among others, could be involved in the increased resistance/tolerance of CAR strain against carvacrol or heat. Further research is needed in order to characterize the genes of this region and their role in the mechanisms of bacterial resistance and tolerance to ICs.

Second, in CIT only, an SNP was confirmed in genome position 1,524,394 bp, which involved the gene *aroC* (Table 2). This gene encodes for chorismate synthase that catalyzes the conversion of 5-enolpyruvylshikimate 3-phosphate to chorismate as a last step in the shikimate pathway, involved in synthesis of aromatic aminoacids [38]. For this reason, according to Foulongne et al. [39], loss of function of *aroC* would lead to a bacterial inability to grow in culture unless aromatic amino acids are provided. Moreover, chorismate is also a precursor of vitamin K and folate. The menaquinone, also known as vitamin K_2_, plays an important role in the electron transport chain and is required for bacterial respiration, where ROS are produced. Thus, if chorismate is not synthesized, the amount of ROS would be reduced [40]. Since ROS are involved in mechanism bacterial death via carvacrol, citral, and limonene [4,5], decrease of ROS production would result in a lower sensitivity against these compounds. However, our CIT strain did not show small colony variants or gentamicin resistance (data not shown), as demonstrated by Wakeman et al. [41] in a mutant strain of *S. aureus* generated with a defect in electron transport.

Chorismate synthase has been extensively studied in order to develop broad-spectrum antimicrobial compounds because the design of appropriate inhibitors for this enzyme could be a plausible way to block multiple pathways essential for the survival of microorganism [42]. In addition, mutants in genes involved in the shikimate pathway, such as *aroC*, have shown an increased susceptibility to the action of some antimicrobial agents, such as ovotransferrin or EDTA, due to defects in cell wall and outer membrane integrity [43]. In CIT, modification of cell envelopes because of the observed SNP in *aroC* might also increase resistance against citral. Even though some studies have related mutations of *aroC* to alterations in cell envelopes, and their implication in the bacterial resistance, no previous research has reported increased resistance against antimicrobial compounds.

Lastly, in the OXLIM strain, one SNP was detected in *rpoB* gene at 588,138 bp genome position (Table 2). This gene encodes for the RNA polymerase β subunit and it is one of the minimal essential genes that has been extensively used for the accurate identification of *Staphylococcus* isolates and for the development of phylogenetic analysis of *S. aureus* [44]. Since rifampicin targets RpoB, mutations located in *rpoB* are closely associated with rifampicin resistance in *S. aureus* [45,46]. However, OXLIM did not show an increased resistance to rifampicin. *rpoB* mutations have also been identified as one of the major contributors to emergence of increased resistance to vancomycin in *S. aureus,* and especially the conversion to vancomycin-intermediate *S. aureus* (VISA) from heterogeneous VISA [47]. 

Such valuable information increases our knowledge of the mechanisms of bacterial inactivation via carvacrol, citral, and (+)-limonene oxide derived from EOs. Even though this research has elucidated the role of some genes in the resistance and tolerance of *S. aureus* against carvacrol, citral, and (+)-limonene oxide, once the whole genome of resistant strains are available, deeper genetic studies are required to acquire further knowledge about the inactivation mechanisms of these natural antimicrobials’ compounds.

## 4. Materials and Methods 

### 4.1. Microorganisms and Growth Conditions

The FPR3757 strain of *S. aureus* USA300 methicillin resistant was provided by Prof. Kolter laboratory (Harvard Medical School, Boston, MA, USA). This strain was isolated from an outbreak in USA [48]. Although, to the best of our knowledge, no food poisoning due to this specific strain has been reported, methicillin-resistant *Staphylococcus aureus* (MRSA) has been commonly isolated from retail meat, with a potential for widespread dissemination in the population [49].

Throughout this investigation, the cultures were kept at –80 °C in cryovials with glycerol. To prepare the broth subcultures one single colony from a plate was inoculated in a test tube with 5 mL of sterile tryptone soya broth (Oxoid, Basingstoke, Hampshire, England) with 0.6% yeast extract added (Oxoid, Basingstoke, Hampshire, England; TSBYE). The inoculated tubes were incubated overnight in aerobic conditions at 37 °C (Selecta Incudigit, Barcelona, Spain) to obtain bacterial subcultures. Two hundred and fifty (250) milliliter Erlenmeyer flasks containing 50 mL of TSBYE were inoculated with these subcultures to a final concentration of 10^5^ colony forming units (CFU)/mL. To reach the stationary growth phase (2 × 10^9^ CFU/mL approx.) bacterial cultures were incubated for 24 h under agitation (130 rpm) at 37 °C (Selecta Rotabit, Barcelona, Spain).

### 4.2. Determination of Minimum Inhibitory Concentration (MIC)

MIC against *S. aureus* USA300 and its derivative strains was determined for citral (95%; Sigma-Aldrich, Steinheim, Westphalia, Germany), carvacrol (95%; Sigma-Aldrich, Steinheim, Westphalia, Germany), and (+)-limonene oxide (97%; Sigma-Aldrich, Steinheim, Westphalia, Germany) with an initial concentration of 10^5^ CFU/mL using the tube dilution method [50,51]. Tested concentrations were: 50, 100, 150, 200, and 250 μL/L of carvacrol or citral; and 500, 750, 1000, 1250, and 1500 μL/L of (+)-limonene oxide. In these experiments, positive controls containing TSBYE inoculated at 10^5^ CFU/mL without ICs, and negative controls containing TSBYE inoculated at 10^5^ CFU/mL with 1500 μL/L of each IC were prepared. Tubes were incubated at 37 °C for 24 h and 130 rpm. MIC was determined as the lowest concentration of IC in the presence of which bacteria showed no visible growth [50].

### 4.3. Isolation of Derivative Strains by Applying Sub-Inhibitory Doses of ICs During Bacterial Growth

The selection of derivative strains of *S. aureus* USA300 was carried out following the procedure described by Kohanski, DePristo, and Collins [1] for bactericidal antibiotics. A *S. aureus* culture grown for 12 h at 37 °C was diluted 1:10,000 in a 250 mL-flask with 50 mL TSBYE and grown at 37 °C and 130 rpm for 3.5 h. After incubation, this culture was diluted 1:3 into TSBYE containing sub-inhibitory concentrations (½ × MIC) of carvacrol, citral or (+)-limonene oxide. Five (5) milliliters of these diluted cultures were grown in tubes at 37 °C and 130 rpm for 24 h. Each day, cells were diluted 1:1000 in a tube containing ½ × MIC concentration of the IC and 5 mL TSBYE and grown at 37 °C and 130 rpm for 24 h. After 10 days, 0.1 mL-samples of the culture were serially diluted in phosphate buffer saline (PBS) and pour-plated in tryptone soya agar (Oxoid, Basingstoke, Hampshire, England) with 0.6% yeast extract added (Oxoid, Basingstoke, Hampshire, England.; TSAYE) to select six colonies. Then, these colonies were grown in TSBYE, and MICs were determined in order to evaluate the direct-resistance against the IC used in the selection process and cross-resistance against other ICs, and to verify the occurrence of stable genotypic modifications.

Complete genomic sequences of the *Staphylococcus aureus* CAR, CIT, and OXLIM strains have been deposited in the NCBI database under the accession numbers CP029030.1, CP029031.1, and CP029032.1, respectively.

### 4.4. Mutagenesis Frequency Evaluation

The mutagenic effect of each IC was determined by calculating the rate of mutants resistant to rifampicin because of point mutations in the *rpoB* gene [11]. Overnight culture of *S. aureus* USA300 was diluted 1:10,000 into 50 mL TSBYE and incubated at 37 °C and 130 rpm for 3.5 h. Afterwards, this culture was diluted 1:3 in tubes containing ½ × MIC of carvacrol, citral, and (+)-limonene oxide and 20 mL fresh TSBYE. This assay was also carried out with norfloxacin (Sigma-Aldrich, Steinheim, Westphalia, Germany) at 2000 mg/L concentration as a positive control. These suspensions were grown at 37 °C and 130 rpm for 24 h (2 × 10^9^ CFU/mL approx. for all suspensions). Samples of the culture were serially diluted in PBS and pour-plated on TSAYE in the presence and absence of 100 mg/L rifampicin (Sigma-Aldrich, Steinheim, Westphalia, Germany). Plates were incubated at 37 °C for 48 h and colonies were counted. Mutation rates were calculated by dividing the number of colonies present in rifampicin plates (mutation events) by the number of colonies present in plates without antibiotic [52].

### 4.5. Evaluation of Increased Bacterial Tolerance 

Prior to the treatments, cultures of WT and the derivative strains were centrifuged for 5 min at 6000× *g* and resuspended in McIlvaine citrate-phosphate buffer of pH 7.0 or 4.0 at a final concentration of 2 × 10^7^ CFU/mL. These pH values were chosen as representative of neutral and acid conditions.

#### 4.5.1. Lethal ICs Treatments

Lethal ICs treatments were carried out in cultures resuspended in 10 mL buffer at both pHs added with 200 μL/L of carvacrol, 2000 μL/L of citral, or 2000 μL/L of (+)-limonene oxide. Treatments were applied at room temperature, except (+)-limonene oxide treatments that were performed at 37 °C. Samples were taken every 2 min up to 20 min at carvacrol treatments and every 10 min up to 120 min at citral and (+)-limonene oxide treatments.

#### 4.5.2. Lethal Heat treatments

Heat treatments were carried out in an incubator (FX Incubator, mod. ZE/FX, Zeulab, Zaragoza, Spain) at 60 °C, and temperature was monitored with a thermocouple (Ahlborn, mod. Almemo 2450, Holzkirchen, Germany). Samples were taken every 2 min up to 8 min.

#### 4.5.3. Lethal PEF Treatments

The PEF equipment (EPULSUS^®^-PM1-10, Energy Pulse System, Lisbon, Portugal) used in this investigation is a Marx generator that can apply monopolar square waveform pulses with a frequency up to 200 Hz, as described by Saldaña et al. [53]. The actual voltage, current, and pulse duration were measured using a high voltage probe (Tektronix, P6015A, Wilsonville, OR, USA) and a current probe (Stangenes Industries Inc., Palo Alto, CA, USA), respectively, connected to an oscilloscope (Tektronix, TDS 220, Wilsonville, OR, USA). As a batch treatment chamber, a cylindrical plastic tube closed with two polished stainless-steel electrodes was used. The distance between electrodes was 0.25 cm, and the electrode area was 2.01 cm^2^. The temperature of the treatment medium was measured and always kept under 30 °C in all experiments performed.

With a sterile syringe, 0.5 mL of the microbial suspension in McIlvaine buffer at pH 7.0 or 4.0 was placed in the batch treatment chamber. Samples were treated at 25 kV/cm with monopolar square pulses of 3 μs (3.75 kJ/kg per pulse) at a repetition rate of 1 Hz, for 15, 30, 45, 60, 75, and 150 μs.

### 4.6. Counting of Viable and Sublethally Injured Cells

Treated samples were appropriately diluted in PBS, and plated onto TSAYE (non-selective medium). Plates were incubated for 24 h at 37 °C. After incubation, the colonies were counted with an improved image analysis automatic colony counter (Protos, Analytical Measuring Systems, Cambridge, United Kingdom). In order to detect sublethal damage at the cytoplasmic membrane [12], some of the treated samples were also plated in the selective medium TSAYE added with 14% NaCl (Panreac, Barcelona, Spain). This concentration of NaCl was the maximum non-inhibitory concentrations for untreated cells as previously determined. Plates with selective media were incubated for 48 h at 37 °C. Bacterial inactivation was calculated by the difference in log_10_ counts before and after the lethal treatments. The proportion of sublethally injured cells was expressed as the difference in log_10_ counts determined in the non-selective and in the selective media.

### 4.7. Genome Sequencing and SNP Analysis

From an overnight culture of WT and derivative strains of *S. aureus* USA300, genomic DNA (gDNA) was extracted using a gDNA kit for extraction and purification (GeneJET Genomic DNA, Thermo Scientific, Waltham, MA, USA). Solexa technology was used to sequence gDNA of the four strains on an Illumina genome analyzer Hi-Seq 2500 instrument (Illumina; Fasteris, SA, Geneva, Switzerland). The quality control filtered paired-end reads (17.3 million 100-bp) were mapped on the *S. aureus* USA300 genome sequence (NCBI accession NC_007793.1) using a Burrows–Wheeler Alignment (BWA) tool [54] giving a raw coverage depth of approximately 650-fold. Mapping covered 91.6%, 95.0%, 94.9%, and 95.8% of *S. aureus* USA300 for our WT, CAR, CIT, and OXLIM strains, respectively. The generated consensus of the four strains sequences were then compared to detect single nucleotide polymorphisms (SNPs) and insertions or deletions (InDels) difference using BWA [54] together with the CLC workbench and SamTools [55] (Software can be downloaded from http://maq.sourceforge.net and http://samtools.sourceforge.net.). All detected SNPs and InDels were tested by PCR and Sanger sequencing.

### 4.8. Statistical Analysis

Results for MIC determination, mutation frequency, disk diffusion assay, and lethal treatments were obtained from at least three independent experiments carried out on different working days with different microbial cultures. These results were represented as the mean ± standard deviation, using GraphPad PRISM^®^ program (GraphPad Software, Inc., San Diego, CA, USA). Data were analyzed and submitted to comparison of averages using analysis of variance (ANOVA) followed by *post-hoc* Tukey test and *t*-tests with GraphPad PRISM^®^. Differences were considered significant if *p ≤* 0.05.

## 5. Conclusions

Although the presence of ICs during *S. aureus* growth decreases mutation frequency, stable resistant strains might emerge during extended food preservation treatments with ICs or EOs, representing a risk for food safety and shelf-life. Since the presence of strains with increased resistance and tolerance might represent a challenge for the design of food preservation processes, further research is required in order to determine whether the emergence of resistant strains in the presence of ICs and EOs is a general phenomenon. In addition, the consequences of the appearance of these mutants and strategies to avoid their emergence warrants further study. WGS of mutant strains allow the identification of stable genetic changes likely involved in their increased resistance and tolerance against ICs and heat treatments. These results lay the groundwork for future detailed genetic studies and demonstrate the power of deep sequencing methods for dissection of cellular responses to essential oils and their constituent components and for the design of future strategies for food preservation.

## Figures and Tables

**Table 1:** Minimum inhibitory concentration (MIC; µL/L) of *Staphylococcus aureus* USA300 wild type (WT) and its derivative strains isolated: CAR, CIT, and OXLIM. Each value represents the MIC of the six colonies isolated by each IC. Increases in MIC with regard to WT are shaded.

	WT	CAR	CIT	OXLIM
Carvacrol	100	150	150	150
Citral	150	150	250	200
(+)-Limonene oxide	750	750	1000	1500

**Table 2:** Mutations of *Staphylococcus aureus* USA300 derivative strains verified by Sanger sequencing.

Genome Position	Strain	Mutation	Gene	Locus tag	Description	Change
2,084,675–2,127,700	CAR	Large deletion	Δ (*hlb1–int3)*	SAUSA300_RS10505-SAUSA300_RS10835	Deletion of 65 genes	-
1,524,394	CIT	SNP (A→T)	*aroC*	SAUSA300_RS07395	Chorismate synthase	N187K
588,138	OXLIM	SNP (C→T)	*rpoB*	SAUSA300_RS02820	DNA-directed RNA polymerase subunit beta	A862V

## Supplementary Materials

The following are available online at http://www.mdpi.com/1420-3049/24/1/170/s1, **Table S1.** Zones of growth inhibition (mm) showing antibacterial activity for carvacrol (1 µL), citral (2.5 µL), and (+)-limonene oxide (30 µL) against *Staphylococcus aureus* USA300 wild type and its derivative strains isolated: CAR, CIT, and OXLIM. Each value represents the mean diameter of the inhibition halo ± standard deviation of the six colonies isolated by each IC. Increases in MIC with regard to WT are shaded. **Table S2.** Primers used for verification. **Table S3.** Large deletion of *Staphylococcus aureus* CAR derivative strain.
