# Supplementary material for: Sub-Inhibitory Doses of Individual Constituents of Essential Oils Can Select for Staphylococcus aureus Resistant Mutants
Source: Molecules. 2019 Jan 4;24(1):170. doi: 10.3390/molecules24010170 (PMC6337159; doi:10.3390/molecules24010170)
Supplement: Supplementary file 1 [file molecules-24-00170-s001.pdf]

SUPPLEMENTAL MATERIAL

Disk diffusion assay

The agar disc diffusion method was used as preliminary assay for testing the antibacterial effect of the ICs against *S. aureus* USA300 and its derivative strains. A total of 0.5 mL from a 10^7 CFU/mL bacterial suspension was spread on the surface of TSAYE plates. Sterile filter paper disks (Whatman Grade 1, 6 mm diameter) were individually impregnated with 1, 2.5, and 30 μL of carvacrol, citral, and (+)-limonene oxide, respectively, dissolved in 3% absolute ethanol (Sigma-Aldrich), and then placed on the previously inoculated agar plates. These volumes were previously determined to produce an inhibition halo measurable (data not shown). Plates were kept at 4 °C for 2 h to allow for the diffusion of ICs, and then incubated at 37 °C for 18–24 h. The diameters of the resulting zones of inhibition were measured with a Vernier digital micrometre (accuracy: 0.01 mm) including the diameter of the paper disk. Disks impregnated with 3% ethanol caused no inhibition halos.

Table S1. Zones of growth inhibition (mm) showing antibacterial activity for carvacrol (1 µL), citral (2.5 µL), and (+)-limonene oxide (30 µL) against *Staphylococcus aureus* USA300 wild type and its derivative strains isolated: CAR, CIT, and OXLIM. Each value represents the mean diameter of the inhibition halo ± standard deviation of the six colonies isolated by each IC. Increases in MIC with regard to WT are shaded.

.	WT	CAR	CIT	OXLIM
Carvacrol	20.49 ± 0.31	14.79 ± 0.61*	17.12 ± 0.60*	16.21 ± 0.49*
Citral	25.73 ± 0.37	26.01 ± 0.49^{ns}	19.14 ± 0.21*	19.77 ± 0.53*
(+)-Limonene oxide	16.04 ± 0.54	16.12 ± 0.38^{ns}	14.34 ± 0.67*	11.18 ± 0.29*

^{ns} not significantly different from wild type ($p > 0.05$).
* significantly different from wild type ($p \leq 0.05$).

Table S2. Primers used for verification.

Verification	Sequence F (5'→ 3')	Sequence R (5'→ 3')
**Deleted 43 kb region in CAR**	CTGACAATAGTGCCAAAGCCG	TGGGGTGTACATTCCACACA
	GGGACCCATTAGGGACTCCA	GGCCGAGTACAGGTGTTTGA
**Flanking regions of 43 kb deletion in CAR**	CTGACAATAGTGCCAAAGCCG	GGCCGAGTACAGGTGTTTGA
***aroC* SNP in CIT**	TCGCATTGCTTGTGCGATAC	TTGCAGTCGGTGCCTTATGT
***rpoB* SNP in OXLIM**	TGGTGCAGAAGTAAAAGATGGAGA	TCAAGAACGATACCGCCAGC

Table S3. Large deletion of *Staphylococcus aureus* CAR derivative strain.

Locus tag	Gene	Product	Locus tag	Gene	Product
SAUSA300_RS10505	*hlb-1*	phosphodiesterase	SAUSA300_RS10680		hypothetical protein
SAUSA300_RS10510		hypothetical protein	SAUSA300_RS10685		DUF1381 domain-containing protein
SAUSA300_RS10515		hypothetical protein	SAUSA300_RS10690		hypothetical protein
SAUSA300_RS10520		hypothetical protein	SAUSA300_RS10695	*dut*	dUTP pyrophosphatase
SAUSA300_RS10525	*scn*	staphylococcal complement inhibitor	SAUSA300_RS10700		hypothetical protein
SAUSA300_RS10530	*chp*	chemotaxis inhibitory protein	SAUSA300_RS10705		DUF1024 domain-containing protein
SAUSA300_RS10535		peptidoglycan hydrolase	SAUSA300_RS10710		hypothetical protein
SAUSA300_RS10540	*sak*	staphylokinase	SAUSA300_RS10715		hypothetical protein
SAUSA300_RS10545	*ami*	amidase	SAUSA300_RS10720		hypothetical protein
SAUSA300_RS10550		phage holin	SAUSA300_RS10725	*rusA*	Holliday junction DNA helicase
SAUSA300_RS10555		hypothetical protein	SAUSA300_RS10730		hypothetical protein
SAUSA300_RS10565		DUF2951 domain-containing protein	SAUSA300_RS10735	*dnaD 2*	replication protein DnaD
SAUSA300_RS10570		hypothetical protein	SAUSA300_RS10740	*ssb2*	single-stranded DNA-binding protein
SAUSA300_RS10575		hypothetical protein	SAUSA300_RS10745		MBL fold metallo-hydrolase
SAUSA300_RS10580		hypothetical protein	SAUSA300_RS10750	*recT*	recombinase
SAUSA300_RS10585		phage tail protein	SAUSA300_RS10755		ATPase
SAUSA300_RS10590		phage tail tape measure protein	SAUSA300_RS10760		DUF1108 domain-containing protein
SAUSA300_RS10600		hypothetical protein	SAUSA300_RS10765		hypothetical protein
SAUSA300_RS10605		phage tail protein	SAUSA300_RS10770		DUF1270 domain-containing protein
SAUSA300_RS10610		hypothetical protein	SAUSA300_RS10775		DUF771 domain-containing protein
SAUSA300_RS10615		hypothetical protein	SAUSA300_RS10780		hypothetical protein
SAUSA300_RS10620		phage head-tail adapter protein	SAUSA300_RS10785		oxidoreductase
SAUSA300_RS10625		phage head-tail adapter protein	SAUSA300_RS10790		hypothetical protein
SAUSA300_RS10630		hypothetical protein	SAUSA300_RS10795		DUF2829 domain-containing protein
SAUSA300_RS10635		phage major capsid protein	SAUSA300_RS10800		XRE family transcriptional regulator
SAUSA300_RS10640		Clp protease ClpP	SAUSA300_RS10805		transcriptional regulator
SAUSA300_RS10645		phage portal protein	SAUSA300_RS10810		XRE family transcriptional regulator
SAUSA300_RS10650		terminase large subunit	SAUSA300_RS10815		ATP-dependent helicase
SAUSA300_RS10655		hypothetical protein	SAUSA300_RS10820		hypothetical protein
SAUSA300_RS10660		HNH endonuclease	SAUSA300_RS10825		hypothetical protein
SAUSA300_RS10665		hypothetical protein	SAUSA300_RS10830		toxin MazF
SAUSA300_RS10670		hypothetical protein	SAUSA300_RS10835	*int3*	site-specific integrase
SAUSA300_RS10675		transcriptional activator RinB			
